# The mechanism of lauric acid-modified protein nanocapsules escape from intercellular trafficking vesicles and its implication for drug delivery

**DOI:** 10.1080/10717544.2018.1461954

**Published:** 2018-04-18

**Authors:** Lijuan Jiang, Xin Liang, Gan Liu, Yun Zhou, Xinyu Ye, Xiuli Chen, Qianwei Miao, Li Gao, Xudong Zhang, Lin Mei

**Affiliations:** a School of Life Sciences, Tsinghua University, Beijing, PR China;; b School of Pharmaceutical Sciences (Shenzhen), Sun Yat-sen University, Guangzhou, PR China;; c The Affiliated Hospital of Guilin Medical College, Guilin, PR China;; d Joint Department of Biomedical Engineering, University of North Carolina at Chapel Hill and North Carolina State University, Raleigh, NC, USA

**Keywords:** Lauric acid, protein nanocapsules, endocytosis, autophagy, drug delivery

## Abstract

Protein nanocapsules have exhibited promising potential applications in the field of protein drug delivery. A major issue with various promising nano-sized biotherapeutics including protein nanocapsules is that owing to their particle size they are subject to cellular uptake via endocytosis, and become entrapped and then degraded within endolysosomes, which can significantly impair their therapeutic efficacy. In addition, many nano-sized biotherapeutics could be also sequestered by autophagosomes and degraded through the autolysosomal pathway. Thus, a limiting step in achieving an effective protein therapy is to facilitate the endosomal escape and auto-lysosomal escape to ensure cytosolic delivery of the protein drugs. Here, we prepared a protein nanocapsule based on BSA (nBSA) and the BSA nanocapsules modified with a bilayer of lauric acid (LA-nBSA) to investigate the escape effects from the endosome and autophagosome. The size distribution of nBSA and LA-nBSA analyzed using DLS presents a uniform diameter centered at 10 nm and 16 nm. The data also showed that FITC-labeled nBSA and LA-nBSA were taken up by the cells mainly through Arf-6-dependent endocytosis and Rab34-mediated macropinocytosis. In addition, LA-nBSA could efficiently escape from endosomal before the degradation in endo-lysosomes. Autophagy could also sequester the LA-nBSA through p62 autophagosome vesicles. These two types of nanocapsules underwent different intracellular destinies and lauric acid (LA) coating played a vital role in intracellular particle retention. In conclusion, the protein nanocapsules modified with LA could enhance the protein nanocapsules escape from intercellular trafficking vesicles, and protect the protein from degradation by the lysosomes.

## Introduction

Nanomedicine refers to the use of nanotechnology for diagnosis, monitoring, prevention and treatment of various diseases (Cheng et al., 2011). Various nanocarriers for diagnosis and treatment based on degradable polymers and non-degradable materials (e.g. polymeric nanoparticles, liposomes, micelles, solid lipid nanoparticles, carbon nanotubes, gold nanoparticles, silica nanoparticles, and dendrimers) have been developed (Cheng et al., 2011, 2017a,b). Proteins have the most dynamic and diverse role of any macromolecule in the body, catalyzing biochemical reactions, forming receptors and channels in membranes, providing intracellular and extracellular scaffolding support, and transporting molecules within a cell or from one organ to another (Leader et al., 2008). Consequently, intracellular delivery of functional proteins has significant therapeutic implications in biological applications, including disease therapies, vaccination, and imaging (Tao et al., 2017). In recent years, a novel delivery platform based on nanocapsules, which consists of a protein core and a thin permeable degradable polymeric shell was developed to deliver proteins to target cells, where the platform break down their shells, enabling the core protein to be active once inside the cells (Yan et al., 2010). However, the poor stability and low cellular permeability of the proteins make protein therapy difficult to apply clinically (Richard et al., 2003). Despite considerable improvements in drug delivery, the development of methods for efficient and specific delivery of targeted therapeutic agents still remain an issue in biological treatments such as gene and protein therapy. The endocytic pathway is the major uptake mechanism of cells and any biological agents, such as DNA, siRNA, and proteins. These agents become entrapped in endosomes or autophagosomes and are degraded by specific enzymes in the lysosome. Thus, a limiting step in achieving an effective biological based therapy is to facilitate the endosomal escape and auto-lysosomal escape to ensure cytosolic delivery of the therapeutics (Varkouhi et al., 2011; Wang et al., 2013).

Lauric acid (LA), a saturated MCFA with a 12-carbon atom chain which makes it hydrophobic, is the primary fatty acid of coconut oil, with the presence of approximately 45–53%. In this study, we incorporated LA into the BSA nanocapsules to design a novel LA-modified BSA nanocapsules to help them escape from the endosome/lysosome and to enable the abundant release of protein into the cytoplasm to increase its bioavailability (Sun et al., 2017). There is literature showing that buffer ability is critical for the NPs endosome/lysosome escape, this buffering capacity results in a ‘proton sponge effect’ and destabilizes the membranes of the endosome/lysosome in the acidic environment and allows the NPs or complexes to escape from the endosome/lysosome rapidly (Wen et al., 2012; Zhang et al., 2017). According to the report, by using the anionic surfactant sodium lauryl-dioxyethylene sulfate and the zwitterionic surfactant cocamidopropyl betaine, in combination with medium chain fatty acids, it is possible to create interfaces with a high dilatational modulus. They hypothesized that mixed micelles of both types of surfactants and fatty acids were formed that transported fatty acids to the interface, where they formed a condensed layer, leading to a high modulus. If a similar mechanism would occur when fatty acids are present in a crude product containing oligofructose fatty acid esters, the fatty acids in the crude product could contribute to the surface properties (Golemanov et al., 2008). So, endosomal escape after endocytosis is a critical step for protein-based agents to exhibit their effects in the cytosol of cells. LA has been associated with certain health benefits of coconut oil intake (Rosamaria et al., 2017). For example, LA has beneficial effects on the cardiovascular system due to its ability to increase the high-density lipoproteins and to reduce the blood pressure and heart rate in both normotensive and hypertensive rats. However, LA has been shown to elicit diverse actions in various tissues, including a potent antimicrobial property and inhibitory effects in colon cancer cells (Dayrit, 2015; Rosamaria et al., 2017). In addition, LA could also trigger antiproliferative and pro-apoptotic effects in both breast and endometrial cancer cells (Rosamaria et al., 2017).

The nanocarriers must enter the cells through inner membrane vesicle system and release the drugs to the target cells to execute their missions. The inner membrane vesicle system is a complex transport system that includes endocytosis, exocytosis, and autophagy, which is responsible for the transport of various macromolecules between different organelles in the cells (Lu et al., 2011; Mizushima & Komatsu, 2011). A nanoparticle placed in the external milieu of a cell can interact with the exterior of the plasma membrane, which can lead to this nanoparticle entry inside the cell through a process termed ‘endocytosis’ which involves multiple stages (Sahay et al., 2010). The approach is based on the proteins involved in different endocytic pathways, which is classified as clathrin-dependent endocytosis clathrin-independent endocytosis (Conner & Schmid, 2003). The clathrin-independent pathways are further classified as caveolae-mediated endocytosis, clathrin- and caveolae-independent endocytosis and macropinocytosis. Clathrin- and caveolae-independent pathways are sub-classified as Arf6-dependent, flotillin-dependent, Cdc42-dependent, and RhoA-dependent endocytosis (Sahay et al., 2010). Autophagy is initiated from an isolated membrane, which engulfs the cytosolic components including long-lived or aggregated prone proteins, superfluous or damaged organelles, and invaded pathogens to form bilayer vesicles (autophagosomes) for degradation (Yang & Klionsky, 2010; Mei et al., 2014). After being activated in response to a variety of chemotherapeutic drugs, autophagy decreases their therapeutic effects and resists cell death. Therefore, autophagy inhibitors such as chloroquine (CQ) have been used to combine with diverse chemotherapeutic drugs and shown to enhance tumor cell killing (Maycotte et al., 2012). Autophagy functions as a tumor suppression mechanism by removing damaged organelles/proteins and limiting cell growth and genomic instability, our previous research has shown that the autophagosomes can sequester the endolysosome escaped nanoparticles into the double membrane vesicles and transport them to lysosomes for degradation, which hinders the advantages of nanoparticles applied for intracellular drug delivery (Zhang et al., 2014).

Rab GTPases are a large family of small GTPases that control membrane identity and vesicle budding, uncoating, motility and fusion through the recruitment of effector proteins, such as sorting adaptors, tethering factors, kinases, phosphatases, and motors (Stenmark, 2009). In humans, there are more than 60 members of the Rab family that are localized to distinct intracellular membranes (Stenmark, 2009). To investigate in detail the trafficking pathways of protein nanocapsules, we used these Rab proteins as vesicle markers to track the intracellular travel pathway of nanocapsules. Here, we prepared a protein nanocapsules based on BSA (nBSA) and modified the nanocapsules with a bilayer of lauric acid (LA-nBSA) to investigate the escape effects from the endosome and autophagosome.

## Materials and methods

### Materials

Lauric acid, bovine serum albumin (BSA), and dimethyl sulfoxide (DMSO) were purchased from Sigma-Aldrich (St. Louis, MO). Dulbecco’s modified Eagle medium (DMEM) and fetal bovine serum (FBS) were obtained from Gibco BRL (Gaithersburg, MD). N-acryloxysuccinimide (NAS), acrylamide (AAm), N,N-methylene bisacrylamide (BIS), ammonium persulfate (APS), N,N,N′,N′-tetramethylethylenediamine (TEMED) and fluorescein isothiocyanate (FITC) were from Aladdin Industrial Co. Ltd. (Shanghai, China). N-(3-Aminopropyl) methacrylamide hydrochloride was purchased from Polymer Science (Monticello, IN). Lipofectamine 2000 was purchased from Life Technologies (Carlsbad, CA). DAPI and Lyso-Tracker Red were from Beyotime Biotechnology (Shanghai, China), Antibodies against LC3, Arf-6, Flotillin, Cdc42, RhoA, P62, EEA1, clathrin, and caveolin were from Cell Signaling Technology, Inc. (Danvers, MA). Antibody against β-actin was obtained from Abmart, Inc. (Shanghai, China). All other materials were commercially available and used as received.

### Synthesis of nBSA

BSA is one of the natural rigid globular proteins with ca. 51% of α-helix secondary structure (Yan et al., 2010). The BSA nanocapsules (nBSA) that consist of a single-protein core and thin polymer shell anchored covalently to the protein core through *in situ* polymerization were synthesized according to a previously reported method (Tao et al., 2017). In brief, firstly, BSA (20 mg) in NaHCO_3_ buffer (4 mL, pH 8, 50 mM) was reacted with NAS (0.5 mg) in 50 mL DMSO for 2 h at room temperature. Then, the reaction solution was thoroughly dialyzed against phosphate buffer (pH 7.0, 20 mM). Using acryloylated BSA solution (10 mg, 1 mg/mL), radical polymerization from the surface of the acryloylated protein was initiated by adding APS dissolved in deoxygenated and deionized water and TEMED into the test tube. A specific amount of N-(3-aminopropyl) methacrylamide (Apm), AAm, and BIS (molar ratio BSA/Apm/AAm/BIS/Aps/TEMED =1/500/2500/300/300/1200) dissolved in deoxygenated and deionized water was added to the test tube over 60 min. The reaction was allowed to proceed for another 60 min in a nitrogen atmosphere. Finally, dialysis was used to remove monomers and initiators. The unmodified BSA was removed using ion exchange chromatography.

### Synthesis of lauric acid-modified BSA nanocapsules

Immobilization of LA onto the nBSA was performed following the previous literature with slight modification (Varkouhi et al., 2011). Briefly, nBSA (100 mg) was dissolved in 10 mL of deionized water. Lauric acid (9.1 g) was dissolved in 3 mL of ethanol. EDC (26.1 mg) was added dropwise into the LA/ethanol solution while stirring at room temperature. After 10 min, NHS (15.6 mg) was added dropwise into the EDC/LA solution while stirring at room temperature for 1 h. The NHS-activated LA solution was added dropwise into the nBSA solution while stirring at room temperature for 24 h. After 24 h, the LA-modified nBSA was precipitated with 80% ethanol. The precipitated material was dried at room temperature and then dissolved in 10 mL of distilled water. The resulting solution was dialyzed against distilled water for 2 d by using a dialysis membrane to remove the unreacted materials, and then lyophilized.

### Characterization of protein nanocapsules

The particle size and zeta potential of protein nanocapsules were measured by Malvern Zetasizer Nano ZS90 (Malvern Instruments, Malvern, UK), and the surface morphology of protein nanocapsules was detected using transmission electron microscopy (TEM, Philips EM120 TEM at 100,000×, Amsterdam, Netherlands). Gel retardation assay was performed with Image Quant LAS4000 after agarose gel electrophoresis in 0.7% agarose gels.

### Cell culture

MCF-7 cells were obtained from Cell Bank of Chinese Academy of Science (Shanghai, China) and cultured in DMEM supplemented with 10% FBS and 1% penicillin–streptomycin (v/v).

### Plasmid and transfection

The EGFP-LC3 and DsRed-LC3 plasmids were created in our laboratory. The DsRed-Rab7 and DsRed-Rab34 were from Addgene (Cambridge, MA). All plasmids were confirmed by automated DNA sequencing. Cells were transfected with the plasmids using Lipofectamine 2000 according to the manufacturer’s instructions.

MCF-7 cells (20,000 cells/well, 12-well plate) were seeded the day before adding the protein nanocapsules. Before the experiment, the medium was then replaced with 0.4 mL of fresh medium. After that, 50 nM FITC-labeled nanocapsules were added into cell medium and incubated at 37 °C for 12 h.

### Cellular uptake of nBSA

MCF-7 cells were seeded into 12-well plates at a density of 2 × 10^4^ cells/well in DMEM containing 10% FBS and allowed to adhere overnight. FITC was used as a model fluorescent molecule and was formulated in nBSA and LA-nBSA. Non-transfected or DsRed-Rab7 and DsRed-Rab34 were incubated with 1 mg/mL FITC-labeled LA-nBSA and nBSA at 37 °C for 4 h. For lysosome detection, the cells were incubated with Lyso-Tracker Red for 1 h. After incubation, the MCF-7 cells in each group were washed with PBS twice. Subsequently, the MCF-7 cells were fixed in 4% formaldehyde for 10 min, and stained cell nuclei with DAPI for 10 min. Then, confocal microscopy was performed with a FLUO-VIEW laser scanning confocal microscope (Olympus, FV1000, Olympus Optical, Tokyo, Japan) in sequential scanning mode using a 60–100× objective. The operation processes were similar to those reported in the literature (Zhang et al., 2014).

### Autophagy assays

Cells were transfected with EGFP-LC3 under the indicated conditions and then fixed in 4% paraformaldehyde. The percentages of cells with fluorescent dots representing EGFP-LC3 translocation were counted by confocal microscopy as described previously (Zhang et al., 2014).

LC3II protein level was detected using an anti-LC3 antibody. Immunoblotting analysis was performed as previously described (Hoyer-Hansen et al., 2007; Liang et al., 2015). In brief, cell lysates were resolved on 12% SDS-PAGE and analyzed by immunoblotting using an LC3 antibody, followed by enhanced chemiluminescence (ECL) detection (Thermo Scientific, Waltham, MA).

### Immunofluorescence assay

Confocal laser scanning microscopy (Fv1000, Olympus Optical, Tokyo, Japan) was used to assess the intracellular trafficking of nanocapsules. MCF-7 cells were plated into glass-bottom dishes at the density of 2 × 10^4^ cells per dish. Briefly, the cells were incubated with the following primary antibodies: EEA1, clathrin, LC3, Arf-6, flotillin, Cdc42, RhoA, P62, EEA1, and caveolin. TRITC and FITC labeled secondary antibodies were used to detect the primary antibodies.

### Statistical methodology

All results are reported as the mean ± SD of three independent experiments. Comparisons were performed using two-tailed paired Student’s *t* tests (*p < .05, **p < .01, ***p < .001).

## Results and discussion

### Preparation and characterization of lauric acid-modified BSA nanocapsules

**Figure 1.**
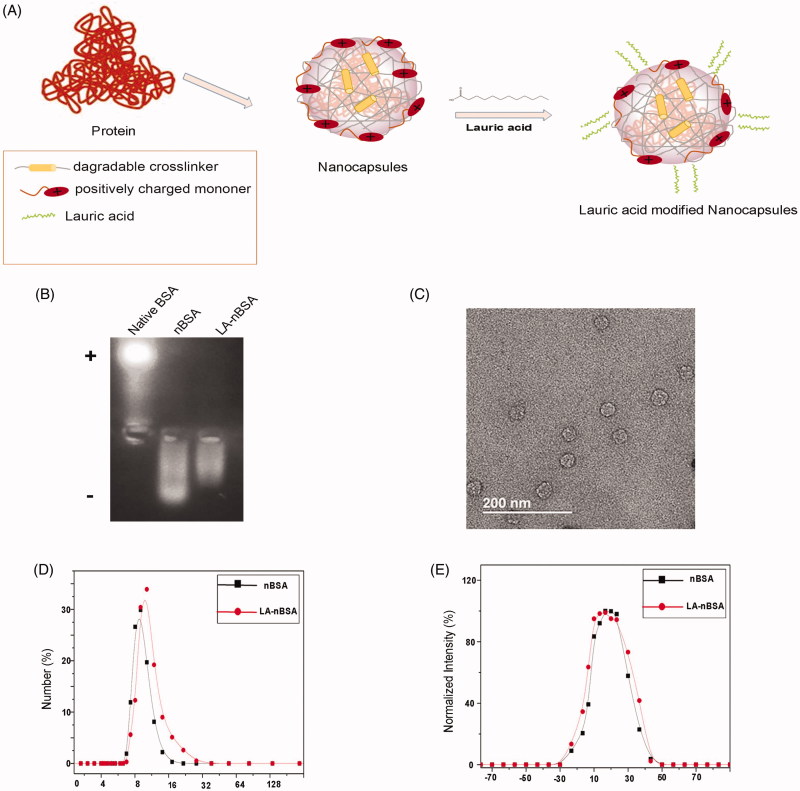
(A) Schematic diagram showing the synthesis of LA-nBSA. (B) Agarose gel analysis of BSA, nBSA, and LA-nBSA. (C) TEM image of LA-nBSA. (D) Particle size distribution and (E) zeta potential of nBSA and LA-nBSA. Scale bars: 50 nm.

In this study, we prepared an LA-modified protein nanocapsule delivery system. The protein delivery platform, which were usually consisted of BSA cores of 5–10 nm in diameter and appropriate coatings. Figure 1(A) is a schematic illustration of the preparation of LA-nBSA. The gel electrophoresis results indicated that the charge of nBSA and LA-nBSA were opposite to that of native BSA. Native BSA had a negative charge, whereas nBSA and LA-nBSA had positive charges, which demonstrates that BSA was completely encapsulated. Meantime, we find that the possess of positive charge of LA-nBSA was less than nBSA, this can be interpreted as for the reduction of the positive amino group in the surface of the nBSA, showing that the nBSA was successfully modified by LA (Figure 1(B)). As shown in the TEM image (Figure 1(C)), the LA-nBSA was spherical, that was in agreement with the nBSA which had a diameter of approximately 20 nm. The size distribution of nBSA and LA-nBSA analyzed using dynamic light scattering (DLS) present a uniform diameter centered at 10 nm and 16 nm (Figure 1(D)). The increase in the particle size was attributed to the presence of LA on the surface of nanoparticles, suggesting the successful modification of LA. The larger size in the TEM images can be attributed to the thickness of the amorphous or surfactant layer on the surface of the nanocapsules. Furthermore, the zeta potential of nBSA and LA-nBSA was measured to be 5.28 ± 0.43 mV and 3.25 ± 0.34 (Figure 1(E)), mainly due to the residual amine groups in the polymer vectors, the experimental results are consistent with the Gel. The positive charge could help protein nanocapsules internalize quickly into cancer cells. The yield of the protein nanocapsules was higher than 95%.

### Endocytosis pathway of nBSA and LA-nBSA

The nanocarriers must enter the cells through inner membrane vesicle system and release the drugs to the target cells to execute their missions. Endocytic pathways represent routes to the insides of cells that are used for a number of cellular processes. It is known that a particular cell type may utilize several different pathways for these and other cellular functions. Thus, the ways in which they are linked together and separated from each other, and the way they interact with other membrane trafficking events are complex.

The trafficking of these molecules is therefore more complex as they can diffuse through the cytoplasm as well as traffic via vesicular and tubular intermediates. Here, we shall principally consider those molecules which enter the cell by endocytosis. Nanomedicines can employ multiple pathways for cellular entry, which are currently insufficiently understood. At least four morphologically distinct pinocytic pathways have been characterized: clathrin-mediated endocytosis, caveolae-mediated endocytosis, macropinocytosis, and clathrin/caveolae-independent endocytosis (Sahay et al., 2010). They differ in the composition of the coat (if any), in the size of the detached vesicles, and in the fate of the internalized particles (Conner & Schmid, 2003). The clathrin-independent pathways are further classified as caveolae-mediated endocytosis, clathrin- and caveolae-independent endocytosis. Clathrin- and caveolae-independent pathways are sub-classified as Arf6-dependent, flotillin-dependent, Cdc42-dependent and RhoA-dependent endocytosis and macropinocytosis (Sahay et al., 2010). To detect how the LA-nBSA and nBSA enter the cells, MCF-7 cells were treated with FITC-labeled LA-nBSA and FITC-labeled nBSA for 20 hours. We observed many FITC-positive vesicles containing LA-nBSA and nBSA within the cells. We then detected the localization of the FITC-positive vesicles with clathrin, caveolin, Arf-6, flotillin, Cdc42, and RhoA positive vesicles. We found that FITC-LA-nBSA positive vesicles co-localized with Arf-6 positive vesicles, but not with clathrin, caveolin, flotillin, Cdc42, or RhoA positive vesicles (Supplementary Figure 1). Similarly, the FITC-nBSA positive vesicles co-localized with Arf-6 positive vesicles, but not with clathrin, caveolin, flotillin, Cdc42, or RhoA positive vesicles (Supplementary Figure 1). These data indicated that the cellular uptake of LA-nBSA mainly through Arf-6-dependent endocytosis. Meanwhile, we found that the co-localization with Arf-6 of LA-nBSA was less than that of nBSA, indicating that the LA-nBSA may be released from endolysosomal entrapment and released to the cytosol. The reason for this is that the surface properties of the nanocapsules are changed due to the modification of the LA.

**Figure 2.**
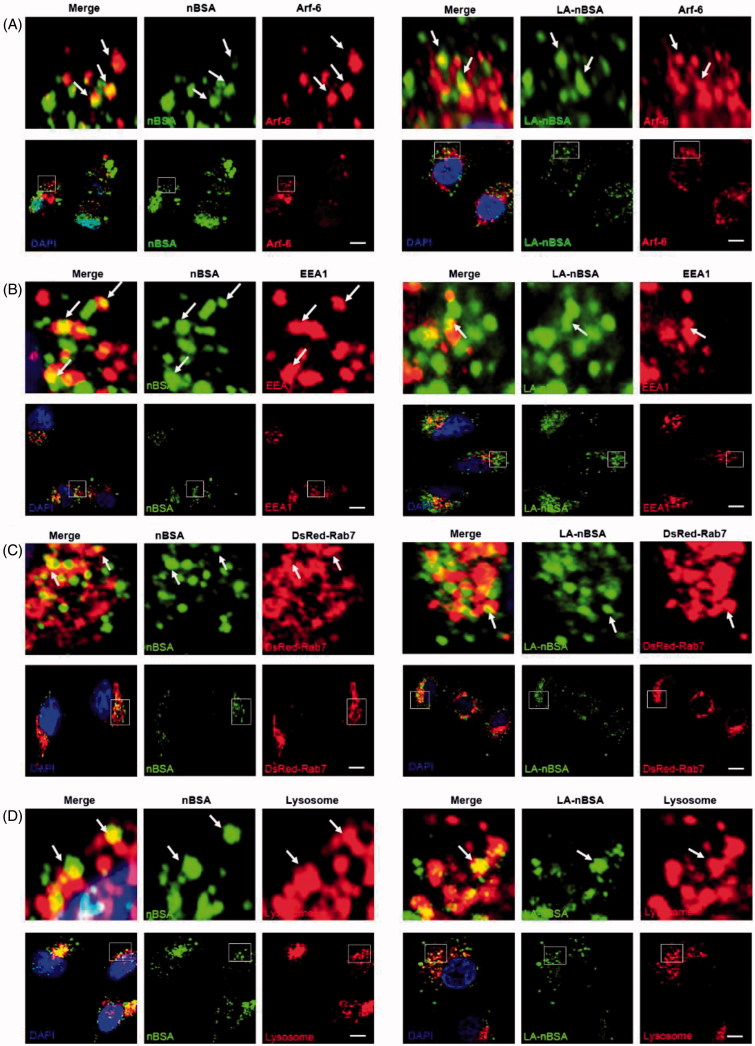
The LA-nBSA enters the cells through Arf-6-dependent endocytosis. (A,B) Confocal images of MCF-7 cells treated with 1 mg mL^–1^ FITC-labeled nBSA and FITC-labeled LA-nBSA for 20 h. Arf-6 and EEA1 were detected with primary antibody against Arf-6 and EEA1, respectively. (C) DsRed-Rab7 transfected MCF-7 cells and then treated with 1 mg mL^−1^ FITC-labeled nBSA and LA-nBSA for 20 h, respectively. (D) For lysosome detection, the MCF-7 cells were treated with 1 mg/mL FITC-labeled nBSA and LA-nBSA for 20 h, respectively, and then co-treated with Lyso-Tracker Red probes for 1 h. Scale bars: 10 μm.

As we all know, the classic endocytosis pathways include early endosomes (EEs), late endosomes (LEs), and lysosomes. EEA1 have been widely used as a marker of EEs, whereas Rab7 is a marker of LEs (Ozeki et al., 2005). Therefore, we used EEA1 to label the EEs and DsRed-Rab7 to label LEs. After that, MCF-7 cells that were transfected with the vector and DsRed-Rab7 were treated with FITC-labeled LA-nBSA and nBSA for 20 h. We found that both vesicles co-localized with EEA1-labeled EEs (Figure 2(B)). In addition, the protein nanocapsules also co-localized with DsRed-Rab7 positive LEs (Figure 2(C)). Lyso-Tracker Red probes were used to detect lysosomes. As expected, both vesicles co-localized with the lysosomes (Figure 2(D)). Moreover, we also found the merge of LA-nBSA was a little fuzzy than the merge of nBSA. It can be demonstrated that the LA-nBSA could escape from the lysosomal degradation more easily than the nBSA. These data demonstrated that the protein nanocapsules were taken up by the cells through Arf-6-dependent endocytosis, transported to LEs and EEs, and finally degraded through the classic endocytosis pathway. However, LA-nBSA was able to penetrate the plasma membrane and enter the cytosol more easily than the nBSA, which tend to be entrapped in endocytic vesicles and co-localize with the endosomes.

**Figure 3.**
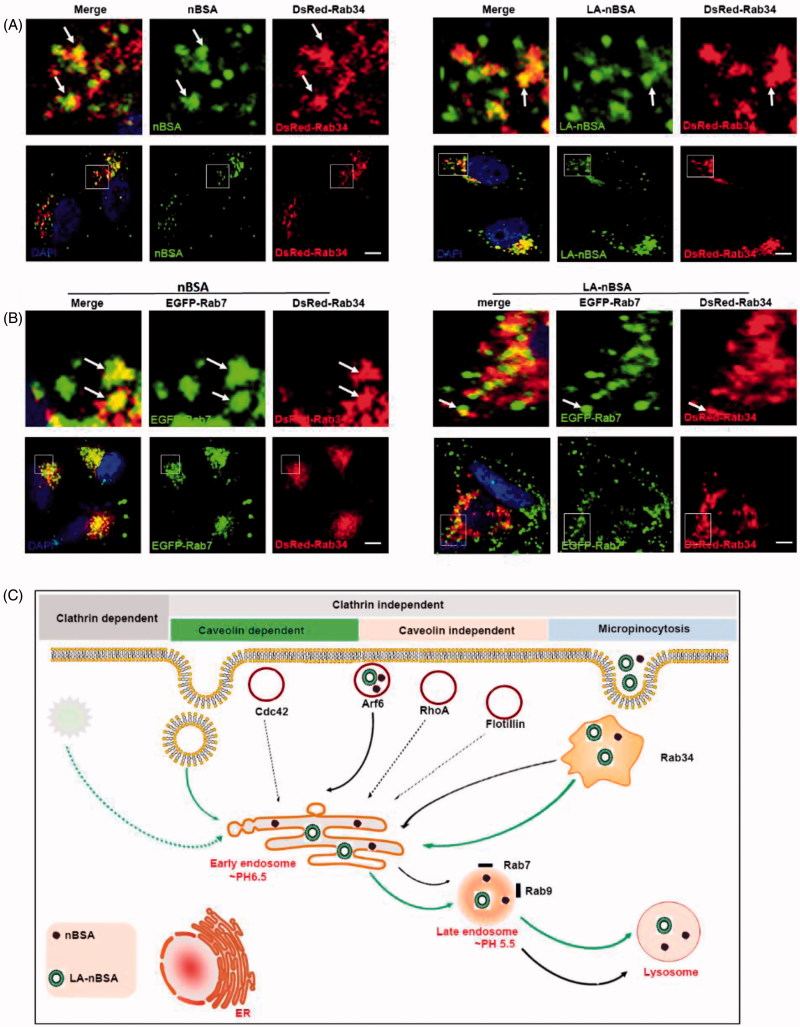
The LA-nBSA was incepted by the cells through macropinocytosis-LEs-lysosome pathway. (A) DsRed-Rab34 transfected MCF-7 cells were then treated with 1 mg mL^−1^ FITC-labeled nBSA and FITC-labeled LA-nBSA for 20 h, (B) DsRed-Rab34 and EGFP-Rab7 co-transfected MCF-7 cells were then treated with 1 mg mL^−1^ nBSA and LA-nBSA for 20 h. Scale bars: 10 μm. (C) Schematic representation of the pathways that nBSA and LA-nBSA enter the cells.

In addition, Rab7 has also been reported to be involved in the endocytosis pathway (Eggenschwiler et al., 2001; Fuller et al., 2014). Macropinocytosis is a type of clathrin independent endocytosis, which is controlled by Rab34. Thus, Rab34 is a marker of macropinocytosis (Bhuin & Roy, 2014). To investigate the other intra-trafficking pathways of protein nanocapsules, the DsRed-Rab34 transfected MCF-7 cells were treated with FITC-labeled LA-nBSA and nBSA. We observed that Rab34 positive vesicles co-localized well with both vesicles (Figure 3(A)). To investigate the downstream pathway of macropinocytosis, we detected the co-localization between DsRed-Rab34 with the markers of the classic endocytosis pathway (EEA1, EGFP-Rab7, and lysosome). Notably, we found that Rab34 could co-localize with EGFP-Rab7 (Figure 3(B)). Furthermore, we also found that the co-localized number of LA-nBSA with Rab34 was higher than that of nBSA. There appears to be signs of escape of the merge of LA-nBSA, which was agreement with previous results. These data demonstrated that the LA-nBSA can escape from the lysosomal degradation more easily than the nBSA. Furthermore, these data indicated that macropinocytosis (Rab34 positive)-LEs (Rab7 positive)-lysosomes might be a novel endocytosis pathway involved in the turnover of LA-nBSA and nBSA.

### Autophagy pathway of protein nanocapsules

**Figure 4.**
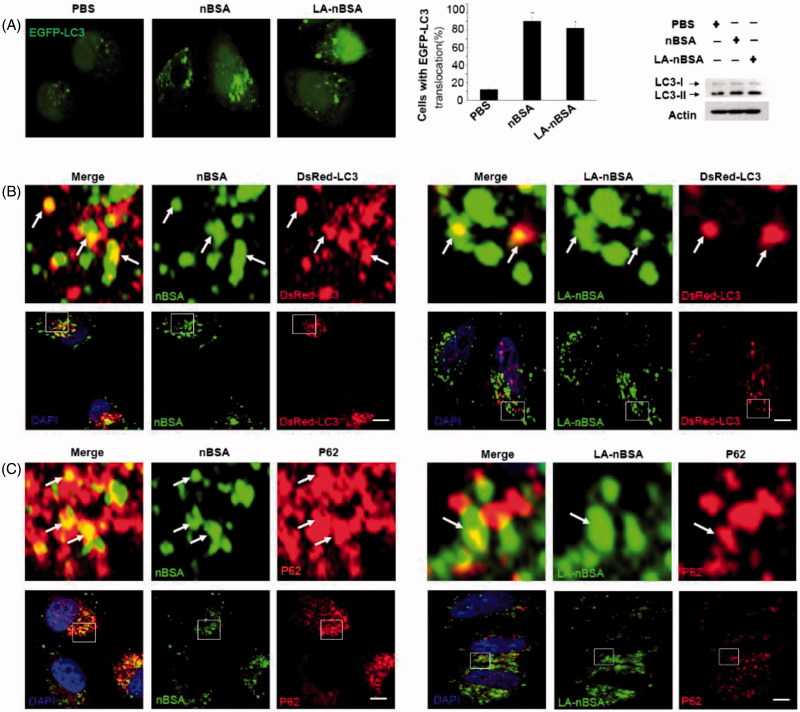
LA-nBSA induces autophagy and is sequestered by the autophagosomes. (A) Representative images and quantification of MCF-7 cells with EGFP-LC3 vesicles (autophagosomes). EGFP-LC3-transfected cells were treated with 1 mg mL^−1^ nBSA and LA-nBSA for 20 h. Scale bars: 10 μm. LC3I/II protein levels were analyzed by western blotting in the MCF-7 cells treated in (A). (B) DsRed-LC3 transfected MCF-7 cells and then treated with 1 mg mL^−1^ FITC-labeled nBSA and FITC-labeled LA-nBSA for 20 h; (C) MCF-7 cells were treated with 1 mg mL^−1^ FITC-labeled nBSA and FITC-LA-nBSA for 20 h, respectively, and then P62 was detected with primary antibody against P62. Scale bars: 10 μm.

**Figure 5.**
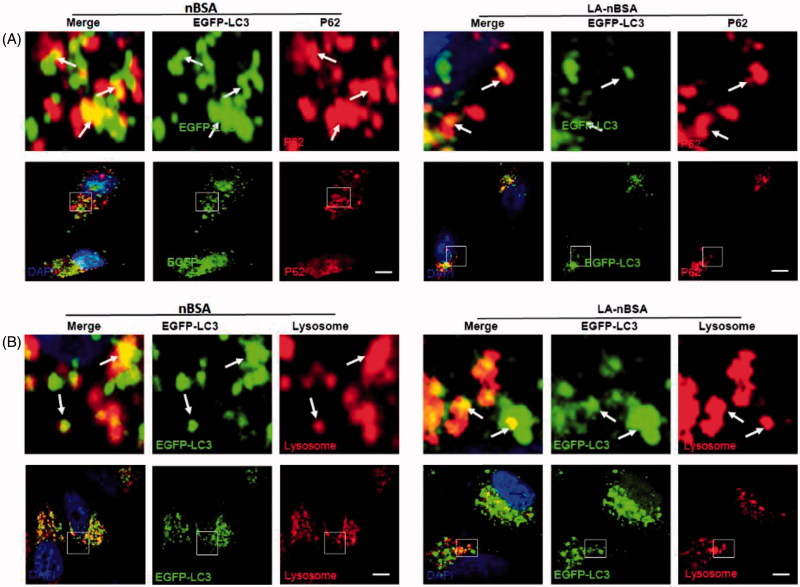
Fluorescent confocal laser scanning microscopic images of LA-nBSA sequestered by the autophagosomes. (A) EGFP-LC3-transfected MCF-7 cells were treated with 1 mg mL^–1^ nBSA and LA-nBSA for 20 h and then P62 was detected with primary antibody against P62. (B) The autophagosomes fuze with lysosomes (arrows). The above images are the enlarged ones in the white collar on the underside images. EGFP-LC3-transfected MCF-7 cells were treated with 1 mg mL^–1^ nBSA and LA-nBSA for 20 h and then co-treated with Lyso-Tracker Red probes for 30 min. Scale bars: 10 μm.

Autophagy or autophagocytosis, which is from the Greek words auto ‘self’ and phagein ‘to eat’, is the basic catabolic mechanism that involves cell degradation of unnecessary or dysfunctional cellular components by the intracellular vesicles called lysosomes (Levine & Kroemer, 2008). In other words, autophagy is an evolutionarily conserved and lysosome-dependent protein degradation pathway. During autophagy, protein aggregates, damaged organelles, and other cellular components may be engulfed by a double-membrane structure to create autophagosome. Then the autophagosome fuses with lysosome and forms an autolysosome. Subsequently, the inner membrane and inside components are degraded by lysosomal hydrolases and released to cytosolic space. Many conditions may induce autophagy, including cellular stresses (such as starvation, oxidative stress, hypoxia, and low pH), aggregated proteins, damaged organelles, and invaded pathogens (Mizushima & Komatsu, 2011). As the components and structure of protein nanocapsules are similar to protein aggregates, autophagy may also sequester these protein nanocapsules (Mei et al., 2014). To measure autophagy, we examined autophagosome (LC3-positive vesicle) formation and LC3II protein levels (Levine & Kroemer, 2008). When autophagy is initiated, LC3 is cut on its C-terminal end to produce the LC3II protein, which is then transferred to the autophagosome. To investigate the relationship between protein nanocapsules and autophagy, MCF-7 cells were transfected with EGFP-LC3. To verify whether the LA-modified protein nanocapsules induce autophagy, EGFP-LC3-transfected MCF-7 cells were incubated with nBSA and LA-nBSA for 20 h. Previous studies by our group have reported that nBSA can produce autophagy (Zhang et al., 2014). As expected, substantial autophagosomes were induced within the cells (Figure 4(A)). LC3-II protein levels were also increased in the cells (Figure 4(A)). The results confirmed that the surface modification of LA did not affect autophagy of protein nanocapsules. To further investigate whether protein nanocapsules could be degraded through the autophagy pathway, we used red fluorescent-labeled LC3 protein (DsRed-LC3) to detect autophagy. DsRed-LC3 transfected MCF-7 cells were treated with FITC-labeled LA-nBSA and nBSA for 20 h. After 20 h incubation, the FITC-labeled nBSA and LA-nBSA were sequestered within DsRed-LC3 positive autophagosomes (Figure 4(B)). P62, which is also named sequestosome 1 (SQSTM1), is a common component of protein aggregates that are found in protein aggregation diseases affecting both the brain and the liver (Bjørkøy et al., 2005). P62 binds to polyubiquitinated proteins and aggregates by oligomerization, and binds to Atg8/LC3 on the autophagosome membrane to target aggregates to autophagosomes for degradation (Pankiv et al., 2007). In addition, P62 is an adapter molecule that selectively recognizes and binds the substrates of autophagy, such as proteins, organelles, and microbes (Stenmark, 2009). As protein nanocapsules are similar to protein aggregates, we assumed that P62/SQSTM1 might mediate the selective autophagy of the protein nanocapsules. Strikingly, as shown in Figure 4(C), we found that the FITC-positive vesicles merged with P62/SQSTM1 in the cells. Significant fluorescence suppression was also observed. In addition, P62/SQSTM1-protein merged with autophagosomes perfectly (Figure 5(A)). Compared to this, we also observed the autophagosomes were then translocated to fuze with the lysosome for degradation. However, quite few yellow dots appeared in the merged image, which indicated that most LA-modified BSA nanocapsules existed within the cytoplasm rather than in the endolysosomes (Figure 5(B)). These data indicated that P62/SQSTM1 selectively captures the nanocapsules and delivers them to autophagosomes. Based on these results, we verified that the modification of LA may facilitate the endolysosome (the fusion of endosome and lysosome) escape of protein nanocapsules, which could protect the nanocapsules from degradation through the autophagy pathway and thus improve the anticancer effects.

## Conclusions

In summary, we successfully developed a novel intracellular protein delivery platform based on protein nanocapsules nBSA and LA-modified nBSA. These nano-carriers may become entrapped in endosomes or autophagosomes and are degraded by specific enzymes in the lysosome. We proposed that the LA in the LA-nBSA may facilitate the cellular uptake of nanocapsules due to its hydrophobic property, and more effectively to delivery hydrophobic drugs than the naked nBSA. A series of experiments demonstrated that FITC-labeled LA-nBSA were mainly internalized by MCF-7 cells through Arf-6-dependent endocytosis and Rab34-mediated macropinocytosis. Moreover, we found that lauric acid-modified protein nanocapsules could efficiently escape from endosomal before the degradation in endolysosomes. Autophagy could also sequester the LA-nBSA through p62 autophagosome vesicles. These experiments also proved that these two types of nanocapsules underwent different intracellular destinies and LA coating played a significant role in intracellular particle retention. The therapeutic efficiency of the clinical used protein and peptide drugs for cancer therapy may be significantly improved by LA modification. To promote the efficiency of protein cytosolic delivery and overcome the problem of insufficient endosomal escape and auto-lysosomal escape challenge, nanocarriers modified with LA will attract highly research interest.
